# MicroRNAs secreted by the parasitic nematode *Brugia malayi* disrupt lymphatic endothelial cell integrity

**DOI:** 10.1371/journal.pntd.0012803

**Published:** 2024-12-31

**Authors:** Hailey Johnson, Stephanie Banakis, Matthew Chung, Elodie Ghedin, Denis Voronin

**Affiliations:** Systems Genomics Section, Laboratory of Parasitic Diseases, Division of Intramural Research, NIAID, NIH, Bethesda, Maryland, United States of America; University of Melbourne, AUSTRALIA

## Abstract

Lymphatic filariasis (LF) is a neglected tropical disease affecting over 51 million people in 72 endemic countries. Causative agents of LF are mosquito-borne parasitic nematodes *Wuchereria bancrofti*, *Brugia malayi*, and *Brugia timori*. The adult parasites impact the integrity of lymphatic vessels and damage valves, leading to a remodeling of the lymphatic system and lymphatic dilation. Chronic infections can develop into severe clinical manifestations, primarily lymphedema, hydrocoele, and elephantiasis. Mechanistic studies on the underlying pathology due to the parasite are necessary to better manage human filariasis. Since parasite molecules, such as microRNAs (miRNAs), can be found in secreted extracellular vesicles (EVs) and are transported between parasite and host cells, we hypothesized that these could also play a role in the development of pathology in LF. In this study, we tested two *B. malayi* miRNAs previously detected *in vitro* in the culture media of microfilarial stages of worms. While one is *Brugia*-specific (bma-miR-5864) and the other nematode-specific (bma-miR-86), both miRNAs are secreted in high abundance. We first examined the *in vitro* response by transcriptomic profiling of human lymphatic endothelial cells to treatment with these miRNAs, which allowed us to identify genes involved in maintaining the integrity of the lymphatic endothelium. We then measured the effect of these miRNAs on the regulation of proteins necessary for cell integrity, demonstrating downregulation leading to a significant increase in the permeability of the endothelium monolayer. With this study we identify parasite miRNAs involved in undermining the integrity of endothelial cells, thus potentially contributing to the development of pathology. These findings could pave the way for a novel treatment strategy where the inhibition of parasite-secreted molecules could slow the progression of LF pathology. From a broader perspective, the miRNAs secreted by filarial parasites could potentially be used in the future for diagnosing and monitoring disease progression or treatment efficacy.

## Author summary

*Brugia malayi* is a species of thread-like worms that reside in the human lymphatic system, leading to the chronic and socioeconomically devastating disease known as lymphatic filariasis. This filarial disease is characterized by significant damage to the lymphatic endothelium, which can result in lymphatic remodeling, disruption of lymphatic flow, and, in severe cases, the development of elephantiasis. To understand the mechanism behind the pathology of lymphatic filariasis, we focused on small regulatory molecules, microRNAs (miRNAs), secreted by the worms and that can interact with lymphatic endothelial cells. We demonstrated that two *B. malayi* miRNAs unique to nematodes, bma-miR-5864 and bma-miR-86, targeted human genes essential for maintaining the integrity of the lymphatic endothelium. Our findings suggest that these bma-miRNAs increase the permeability of the endothelial barrier, potentially causing lymphedema and contributing to lymphatic filariasis pathology. This research provides new insights into the molecular interactions between parasitic worms and the host’s lymphatic system, which could inform therapeutic strategies.

## Introduction

Lymphatic filariasis (LF) is a neglected tropical disease caused by mosquito-borne parasitic nematodes *Wuchereria bancrofti*, *Brugia malayi*, and *B*. *timori*. Over 51 million people are estimated to be infected in 72 endemic countries, while another 882 million remain at risk of infection (WHO, 2024). LF is especially detrimental to communities already facing socioeconomic challenges, urging the need for affordable treatment and improved diagnostic methods to prevent the spread of disease [1,2].

Filarial parasites are transmitted by vector mosquitoes that collect blood containing microfilariae, the first stage of worm development. Microfilariae then develop to infective third-stage larvae (L3) in the vector. During the mosquito’s next feeding, the L3s penetrate the skin of the vertebrate host and migrate to the lymphatics where they develop into adults. Adult parasites can survive in the lymphatic vessels for 8–10 years, releasing millions of microfilariae over their lifetime [3].

The presence of adult parasites is thought to be required for the progression of pathology in humans, the definitive hosts. Infection leads to damaged lymphatic valves and remodeling of the lymphatic vessels because of proliferation of endothelial cells and connective tissues [4,5]. This phenomenon can occur early in infection [6], and, in some cases, in patients without microfilariae circulating in the blood or in patients with negative antigen tests for LF [7], resulting in lymphatic dilation not necessarily limited to the location of worm nests. Observations of both *W. bancrofti* and *B. malayi* human infections show that early-stage lymphatic dysfunction occurs before the onset of visible symptoms of the disease [6].

Chronic infections may lead to severe clinical manifestations, primarily lymphedema, hydrocoele, and elephantiasis, with the transition from lymphangiectasia to chronic disease. Globally, it is estimated that over 40 million people are affected by overt lymphatic disease. The prevalence of hydrocoele in Bancroftian filariasis (caused by *W. bancrofti*) is found to correlate with the intensity of infection, particularly adult worm burden in the lymphatic system [8]. Adult worms have been consistently detected in intrascrotal lymphatics in cases of hydrocoele. Histological data showed that parasitic infection can cause morphological and functional changes to the lymphatic endothelial cells in humans: infection decreased the number of intracytoplasmic vesicles transporting interstitial fluid, culminating in edema in the patient [4,9,10].

There is no indicated treatment for lymphedema caused by filarial infection, and currently most therapeutic interventions focus on the use of antibiotics, such as amoxicillin and doxycycline, for secondary skin bacterial infections [11]. Some murine model studies have shown that doxycycline and minocycline are promising therapeutics for reversing the severity of filarial lymphedema and lymphatic remodeling in *B. malayi* infections. Although tetracyclines end up targeting the bacteria, *Wolbachia*, present as endosymbionts in filarial worms, minocycline and doxycycline mitigate lymphatic damage independently of anti-*Wolbachia* or general antibiotic activity [11].

Elimination of microfilariae via annual treatment with albendazole and ivermectin or diethylcarbamazine (DEC) is the current standard for intervention of disease transmission. In addition to their microfilaricidal properties, annual doses of DEC and albendazole were shown to significantly reduce the proportion of *W*. *bancrofti* in infected children. Following treatment, pediatric cases of Bancroftian filariasis also showed reversed disease pathology, including improved lymphatic flow [6]. Interventions to the host’s early immune response to infection were shown to slow the development of LF pathology [11]. The human lymphatic endothelium is comprised of cells that mediate immune responses and inflammation, and therefore it is an important interface in the pathogenesis of LF [4].

As adult filarial worms reside in the lymphatic system, lymphatic endothelial cells (LECs) interact directly with the filarial worms and with their secreted products. Some of the molecules secreted by the worms include microRNAs (miRNAs). These are a highly conserved group of small, non-coding RNA molecules, generally 20–25 nucleotides long, and found in almost all animal species, including filarial parasites. While they do not encode proteins, miRNAs function as regulators of mRNA transcripts, thus mediating important cellular processes [12,13]. The delivery of miRNAs to host cells can occur via extracellular vesicles (EVs), shown to be secreted by nearly all animal cells and important for transporting molecules between cells and organisms [14]. Previous studies of other parasite species have shown that parasite miRNA delivered to host cells through EVs during infection can modulate host mRNA expression [15–17].

In this study, we tested the hypothesis that *B. malayi* miRNAs can impact cell physiology and contribute to the pathology of lymphatic filariasis (LF). We profiled the response of human LECs (hLECs) to parasite miRNAs with a particular focus on the integrity of the endothelium monolayer. MiRNA-transfected cells significantly downregulated the expression of genes that encode essential proteins for cell adhesion and maintenance of the integrity of the endothelium. We validated that the downregulation of these proteins by parasite miRNAs leads to increased leakage in a monolayer of hLECs.

These findings suggest a novel treatment strategy where the inhibition of parasite-secreted molecules could help manage the progression of LF pathology. For example, specific miRNA inhibitors that bind in a complementary fashion to target miRNA could be developed as prospective therapeutics for LF. Moreover, the miRNAs secreted by parasites could be used in the future for diagnosing and monitoring disease progression or treatment.

## Results

### *B. malayi* bma-miR-5864 and bma-miR-86 affect mRNA expression of genes in hLECs

A previous study demonstrated *in vitro* that *B. malayi* microfilariae (MF) secreted miRNAs that could be detected in the culture supernatant as free-floating molecules or within EVs [16]. We hypothesized that adult worms would also secrete miRNAs into their culture media. Indeed, the MF-secreted miRNAs (bma-miR-100, bma-miR-71, bma-miR-34, and bma-miR-7) that are predominantly detected in EVs were shown to modulate the mTOR pathway in human monocytes (THP-1 cells) [16]. Here we selected two miRNAs that were found to also be highly abundant in the supernatant/EVs of cultured MF and that had no significant homology to human miRNAs: bma-miR-5864 (MIMAT0026363), specific to *Brugia,* and bma-miR-86 (MIMAT0031762), specific to nematodes. miRNA has a seed site mostly situated at positions 2–8 from the 5’ end. Bma-miR-86 has the site from nucleotide position 2 to 8 with an identical sequence (5’-AAGTGAA) found in bma-miR-5864 at positions 3 to 9. Therefore, these two miRNAs can potentially target similar genes/pathways in human cells. Next, we confirmed their presence in the EVs collected from cultured adult *B. malayi* worms using TaqMan RT-qPCR probes specific for bma-miR-5864 and bma-miR-86. Both bma-miRNAs were detected by qPCR (n = 3) in EVs from female (Ct value average 31 for bma-miR-5864 and 27.5 for bma-miR-86) and male worms (Ct value average 33.5 for bma-miR-5864 and 30.6 for bma-miR-86).

We then predicted potential transcript targets for these bma-miRNAs by searching the 3’untranslated region (UTR) of human genes with miRanda, an algorithm that uses a dynamic-programming alignment and thermodynamics to predict mRNA targets for miRNAs [18]. We obtained 7,769 and 3,144 human genes with potential target sites for bma-miR-5864 and bma-miR-86, respectively; 2,748 human genes have seed sites on their UTRs for both miRNAs (S1 Table).

To identify which human genes have altered expression in response to bma-miR-5864 or bma-miR-86, we conducted a transcriptomic analysis using hLECs transfected with mimics of bma-miR-5864, bma-miR-86, or a negative control (NC-5, ThermoFisher). We identified 788 differentially expressed genes (DEGs) in hLECs treated with the bma-miR-5864 mimic compared to the negative control, and 811 DEGs in hLECs treated with the bma-miR-86 mimic (S2 Table). Of these genes, 519 were downregulated in response to treatment with the bma-miR-5864 mimic and 436 in response to the bma-miR-86 mimic.

**Fig 1:**
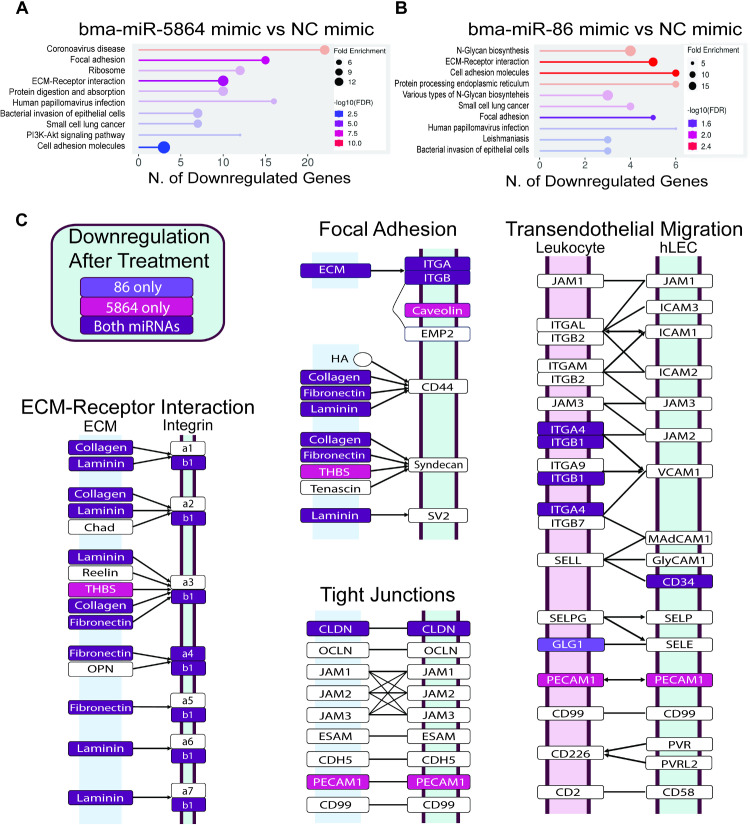
KEGG enrichment analysis of bma-miR-5864 mimic and bma-miR-86 mimic transfected hLECs. **(A)** Top 10 KEGG terms of downregulated genes in bma-miR-5864 mimic transfected hLECs. **(B)** Top 10 KEGG terms of downregulated genes in bma-miR-86 mimic transfected hLECs. Many of the genes in these pathways are downregulated in samples treated with either bma-miR-5864 mimic or bma-miR-86 mimic, while some genes exhibit downregulation specific to one miRNA treatment. **(C)** Details of selected KEGG terms (from A and B) showing downregulated genes in bma-miRNA-86 mimic (light purple boxes) or bma-miRNA-5864 mimic (pink boxes) in transfected hLECs. White boxes indicate genes that showed no significant changes in their expression.

Of the 519 human genes downregulated in bma-miR-5864 mimic-treated cells, 232 genes (44.7%) have predicted seed site(s) for the miRNA on their 3’UTR, while of the 436 human genes downregulated in bma-miR-86 mimic-treated cells, 96 (22%) have predicted seed site(s) (S3 Table). The most significant (the lowest p adjusted value) differentially expressed gene for both treatment groups is *FN1*, which encodes fibronectin 1, a glycoprotein involved in cell adhesion and migration processes [19]. The significant DEGs also include other genes that encode proteins important for cell-to-cell adhesion, such as thrombospondin 1 (*THBS1*) and fibrillin-2 (*FBN2*) (S3 Table) [20,21]. A KEGG enrichment analysis of the downregulated (519 human) genes in response to bma-miR-5864 mimic treatment revealed KEGG terms such as “extracellular matrix-receptor (ECM-Receptor) interactions” and “focal adhesion” to be significantly enriched in treated samples (Fig 1A). Similar results were obtained for bma-miR-86 mimic-treated cells after analysis of the 436 downregulated genes (Fig 1B and 1C). These observations indicate that the bma-miRNAs tested may be compromising the integrity of the hLECs in culture.

The analysis also revealed that ribosomal genes and genes involved in responses to RNA virus infections (“COVID-19 virus” term) were significantly enriched KEGG terms in the treatment groups as compared to the control (NC) (Fig 1A and 1B), indicating that these bma-miRNAs could be eliciting an innate immune response to the presence of excess intracellular RNAs. It suggests that the cellular response is not only to the introduced miRNA, but also to host mRNAs that are fragmented as a result of interference with the bma-miRNAs.

Enrichment analysis of downregulated genes (in bma-miR-5864 mimic treated cells) revealed that one of the most affected pathways (fold enrichment > 11) is ECM-Receptor interaction (S4 Table). This pathway consists of genes (*LAMA3*, *ITGA4*, *FN1*, *COL4A2*, *LAMC1*, *THBS1*, *ITGAV*, *ITGB1*, *COL6A3*, and *COL4A1*) that play essential roles in maintaining the monolayer of endothelial cells. This pathway is also significantly affected in bma-miR-86 mimic treated cells (S4 Table). Of all the downregulated and potential target genes, 22 (bma-miR-5864) and 18 (bma-miR-86) genes encode proteins for cell-to-cell connection, cellular adhesion, and interaction with the extracellular matrix, and are key to supporting the integrity of the lymphatic endothelial barrier which becomes compromised in LF patients.

### Bma-miR-5864 and bma-miR-86 increase permeability of the hLEC cellular monolayer

**Fig 2:**
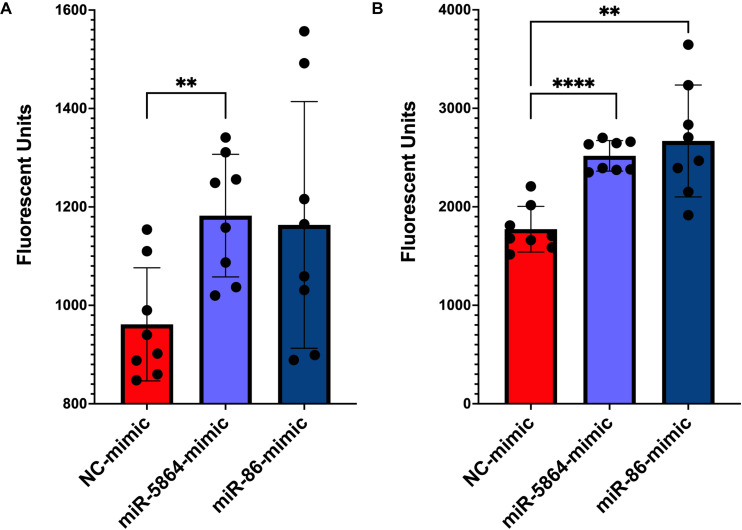
Permeability of hLECs following treatment with bma-miR-5864 and bma-miR-86 mimics. **(A)** Fluorescent signal in wells after 24 hours post-transfection with miRNAs. Bma-miR-5864 mimic transfected samples exhibit significantly higher fluorescence intensity than NC-treated samples. Though not statistically significant (*p*-value = 0.0571), the bma-miR-86 mimic also demonstrates an increase in fluorescence intensity. **(B)** 48 hours post-transfection with miRNAs as compared to NC. The significant increases in fluorescence, respectively, indicate bma-miR-5864 and bma-miR-86 mimics increase the permeability of the cellular monolayer in the lymphatic endothelia cells. ** *p*-value < 0.01 and **** *p*-value < 0.0001.

The bma-miRNAs tested are predicted to target mRNAs encoding genes that are essential for the integrity of the hLEC monolayer. We confirmed that the expression of these genes is downregulated in bma-miRNA treated cells as compared to the negative control. Therefore, we tested the direct effect of parasite miRNAs on monolayer integrity using a permeability assay (Millipore Sigma) following miRNA-mimic transfection. As compared to untreated control samples, we observed that hLECs transfected with the bma-miR-5864 mimic or bma-miR-86 mimic had reduced functionality of cell-to-cell connection proteins and increased permeability of the cellular monolayer *in vitro*. The monolayer of hLECs transfected with bma-miRNAs showed detectable increased permeabilization at 24 hours post-treatment as compared to the NC (miR-5864 mimic, *p*-value = 0.0025; miR-86 mimic, *p*-value = 0.0571) (Fig 2A). After 48 hours, transfected hLECs were nearly twice as leaky as NC-treated cells (miR-5864 mimic, *p*-value < 0.0001; miR-86 mimic, *p*-value = 0.0010, Fig 2B). The monolayer of hLECs transfected with bma-miRNAs mimics showed gaps between cells detectable by bright field microscopy at 48 hours post-treatment as compared to the NC mimic (S1 Fig).

### Bma-miR-5864 and bma-miR-86 modulate host protein expression and distribution in hLECs

**Fig 3:**
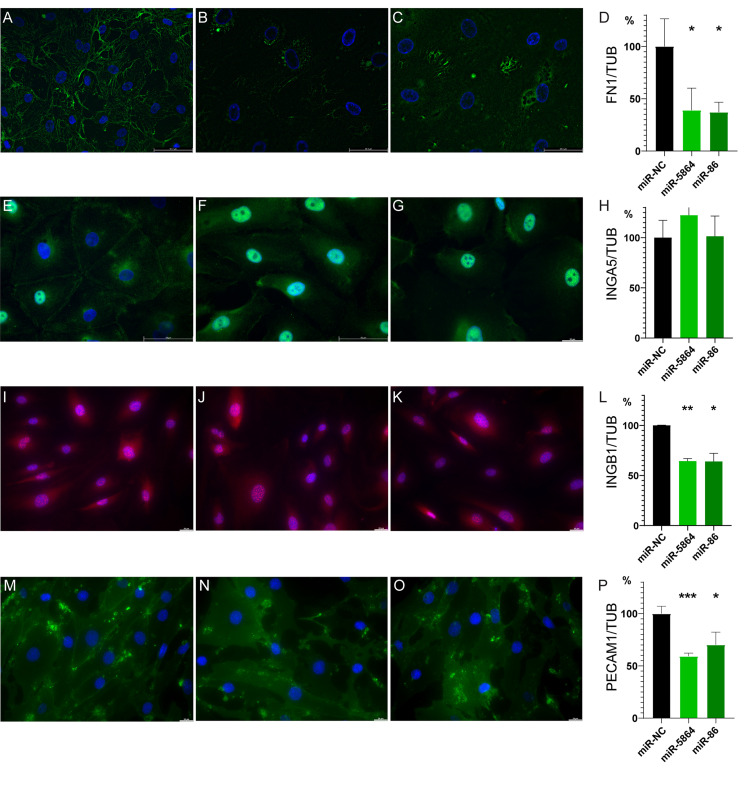
Localization and expression of cell-to-cell connection proteins in bma-miRNA transfected and NC hLECs. **(A)** FN1 (green) observed as a net-like structure in NC cells with nuclei shown in blue (DAPI). (**B** **and** **C)** Reduction of protein expression and absence of FN1 (green) net-like structure in cells treated with bma-miR-5864 mimic (B) and bma-miR-86 mimic (C). (**D)** After 2 days of treatment, both bma-miR-mimics significantly reduced relative expression of the FN1 protein in cells as compared to control cells (miR-NC-mimic, taken as 100% of expression, n = 4). Relative expression was normalized using a signal of αTUB of the same sample. **(E)** ITGA5 (green) expression is localized to cell borders, with occasional presence in the endoplasmic reticulum and cell nucleus (DAPI). (**F** **and** **G)** ITGA5 (green) is mainly present in cell nuclei of cells treated with bma-miR-5864 mimic (F) and bma-miR-86 mimic (G). **(H)** No significant changes to ITGA5 relative protein expression were observed (n = 3). (**I–K)** ITGB1 (red) expression appears similar across treatment groups (I – NC, J – bma-miR-5864, and K – bma-mir-86), with mildly decreased expression observed in parasite miRNA-treated cells (J, K). **(L)** Significant reduction in ITGB1 relative protein expression demonstrated in cells treated with bma-miR-5864 and -86 mimics as compared to control (n = 3). **(M)** PECAM-1 (green) observed on cell borders and occasional presence in the cytoplasm of NC-treated cells. (**N** **and** **O**) Reduction of PECAM-1 on cell borders observed in cells treated with bma-miR-5864 mimic (N) and bma-miR-86 mimic (O). **(P)** PECAM-1 relative protein expression is significantly reduced in both bma-miR-mimic treatment groups as compared to control (taken as 100% of expression, n = 2). Magnification 40x.

To validate that secreted bma-miR-5864 and bma-miR-86 change cell physiology, we studied the localization and expression of cell-to-cell connection proteins in hLECs transfected with the miRNA mimics. In control cells, fibronectin-1 (FN1) forms a net-like structure that is distributed over the bottom of the cells (Fig 3A). Bma-miR-5864 and bma-miR-86 mimics dramatically reduced the expression and disrupted the localization of FN1 (Figs 1C and 3B) in transfected cells as compared to controls. The relative expression of FN-1 (normalized to the expression of alpha tubulin (αTUB) and determined by Western blot) was significantly reduced in transfected cells as compared to the NC (Fig 3D).

Integrins α5 and ß1 (ITGA5 and ITGB1, respectively) serve as proteins that make complexes with FN1 to form the cellular monolayer. Transcriptomic data did not show significant changes in ITGA5 expression. As it is a key protein that works with FN1 and ITGB1, we determined its localization in cells. In NC-treated cells, ITGA5 is typically localized throughout the cellular membrane, especially on the borders between cells (Fig 3E). In some cases, we observed ITGA5 in the nuclei of NC cells. In bma-miRNA-transfected cells, the majority of the signal was observed in the nuclei of hLECs and rarely found on the edges of cells (Fig 3F and 3G). Western blot analysis of ITGA5 relative protein expression revealed no change in the level of proteins in bma-miRNAs transfected vs NC cells (Fig 3H). Transcriptomic data showed significant downregulation of ITGB1 in response to bma-miR-5864 mimic and bma-mir-86 mimic treatment. ITGB1 did not exhibit notable changes in localization in transfected cells as compared to NC cells, however, a small reduction of ITGB1 signal in bma-miR-5864 and -86 mimics treated cells can be observed (Fig 3I–3K). Despite no major visible changes to ITGB1 localization in LECs, relative expression of ITGB1 protein (normalized to αTUB) was significantly decreased in transfected cells as compared to controls (Fig 3L).

To assess disruption of tight junction complexes in hLECs, we looked specifically at the expression of the platelet endothelial cell adhesion molecule-1 (PECAM-1). Using IF microscopy, we observed significant disruption in protein localization in cells transfected with bma-miR-5864 and bma-86 mimics as compared to NC-treated cells (Fig 3M–3O). Clear cellular borders lined with PECAM-1 are demonstrated in control cells while these edges are absent or significantly diminished after exposure to the parasite miRNAs. The relative expression of PECAM-1 was also significantly decreased in parasite miRNA-transfected hLECs (Fig 3P).

## Discussion

Adult parasitic nematodes that reside in the lymphatics cause damage to host lymph vessels and valves, leading to broken barriers between surrounding tissues and lymph. In this study, we investigated the potential role of two secreted *B. malayi* miRNAs in parasite-host interactions and in the pathology of LECs. miRNAs are regulatory molecules that act by binding to target mRNA, thereby silencing the expression of encoded proteins. Previous studies have shown that *Brugia* miRNAs can be found outside the microfilarial stage of the parasite and in secreted EVs in *in vitro* culture [16]. We focused on two highly abundant miRNAs found in Mf-derived EVs, with one specific to *Brugia* (bma-miR-5864) and one specific to nematodes (miR-86). These parasite miRNAs may have unique functions in filarial infection and are less likely to interfere with human miRNA homologues. Though bma-miR-5864 is noted as being *Brugia*-specific, the gene and 200nt surrounding its genomic location were also found in the genome of *Wuchreria bancrofti* and *B. timori*, other filarial worms that cause LF. The sequence was not found to be encoded in the genome of *Onchocerca volvulus*, a filarial worm that resides subcutaneously and causes River Blindness, nor in the genome of the ocular filarial worm, *Loa loa*.

In this study, we first confirmed the presence of bma-miR-5864 and bma-miR-86 in secreted EVs isolated from adult male and female worms (females also produce MF). It is known that parasitic nematode-secreted EVs contain parasite miRNAs that can interact with the host [14]. MicroRNAs secreted from gastrointestinal parasites have been shown to modulate host innate and adaptive immune responses [22]. For example, in *Fasciola hepatica* infection, parasite miRNAs are predicted to target fundamental signaling pathways in the host, including Ras, MAPK, P13K-Akt, and Wnt, along with those involved with the host immune response [23]. As a result, worms are protected from host responses and neutralization. Numerous studies have demonstrated that parasite-derived miRNAs can enter a diverse set of host cells in both *in vitro* and *in vivo* models of infection [24–27]. The filarial nematode *Litomosoides sigmodontis* and various gastrointestinal nematodes (*Heligmosomoides polygyrus*, *Trichuris muris*, *T. suis*, and *Ascaris suum*) secrete miRNAs that incorporate into, and interfere with, host immune cells such as macrophages and T lymphocyte cells [24,25,28]. Additionally, the presence of parasite miRNAs in host lymph nodes has been observed in *L. sigmodontis* and *Schistosoma mansoni* infections [28,29], two species that do not dwell in the lymphatics as *B. malayi* does. Vascular endothelial cells that internalize *S*. *mansoni* miRNAs show responses at the transcriptomic level with decreased expression of genes associated with the complement system and coagulation pathways [26]. Secreted miRNAs from filarial nematodes *B. pahangi, Dirofilaria immitis*, and *O. volvulus* have previously been detected in host blood during infection [30]. With LF, human dendritic cells are known to uptake *Brugia* miRNAs from microfilarial EVs, resulting in downregulation of the mTOR pathway and induction of host autophagy [16].

As adult filarial worms reside in the lymphatic system, LECs are at the interface of host defenses and parasite-secreted molecules. Moreover, human LECs are a major mediator for immune responses, including inflammation, and therefore are important for the pathogenesis of LF [31]. Human lymphatic endothelial cells are used as a model to mimic *in vitro* adult worm infection in the host. Through the study of parasite-hLEC interactions we can gain additional insight as to the role parasite miRNAs play in regulating the host immune response via interference with endothelial barriers.

Our transcriptomic analysis of parasite miRNA-transfected hLECs showed a strong reduction of transcripts that encode proteins important for maintaining the integrity of the endothelial cellular monolayer. Particularly, enrichment analysis of the transcriptome revealed that miRNA transfection significantly suppresses cellular adhesion and cell-to-cell connection pathways, including extracellular matrix-receptor interaction, cellular adhesion, and tight and focal junctions. The loss of function of the genes in these pathways as a result of miRNA interference could contribute to increased lymphatic vessel permeability and the clinical manifestations LF patients experience.

During filarial infection, the endothelial barrier is compromised, resulting in fluid leakage and lymphedema in the affected person. These changes to the host lymphatic system happen very early during infection, though vessel damage is not always localized to the worm nests [6]. Indeed, we observed parasite miRNAs dramatically reduce the hLEC monolayer integrity and increase leakage. We have shown by Western blot that proteins essential for sustaining the lymphatic barrier are downregulated (such as Fibronectin-1 and PECAM-1) or showed displacement by immunofluorescent microscopy (such as ITGA5) in miRNA-transfected hLECs. Fibronectin is the major ligand for the α5β1 integrin complex. Reduction of fibronectin and integrin β1 expression found in this study can result in the relocation of integrin α5 in endothelial cells. This is supported by experiments where knocking out β1 specifically in endothelial cells resulted in severe adhesion and migration alterations, as well as the reduced survival of endothelial cells [32]. The low expression and/or disrupted location of key endothelial barrier proteins is one explanation for dysfunction in the lymphatics.

Other helminths and protozoa have been found to interact with the host’s interfacing layers [33]. Intestinal parasites are especially damaging to the epithelial barrier. *Giardia intestinalis* and *Blastocystis ratti* induce apoptosis in intestinal epithelial cells, thus increasing permeability and compromising the intestinal barrier [34–36]. Increased epithelial permeability was also found in the acute phase of *H. polygyrus* infection in a murine model. These findings were consistent in humans with experimentally acquired *Necator americanus* infection: all study participants exhibited increases in their small intestine permeability during the acute phase of infection [37]. According to our findings, the reorganization of the endothelium can, in part, be stimulated by parasite secreted miRNAs. This observation supports future studies into the role parasite miRNAs play in the pathology caused by helminth infections.

Human vascular and lymphatic vessels are lined with endothelial cells that interact with immune cells and pathogens. Integrins are essential proteins for maintaining cellular adhesion, migration, and survival. These transmembrane glycoproteins are important facilitators of the Extra Cellular Matrix (ECM) and endothelial cell binding, among other roles in angiogenesis [38]. Fibronectin, a matrix forming protein, is a known ligand of dimeric integrins. Together, these proteins mediate the adhesion and migration of LECs, and they are involved in complex signaling cascades influencing gene expression [39]. Little is known about the functional relevance of integrins in lymphangiogenesis, though studies suggest the a5b1 heterodimer plays some role in regulating the process. Due to their roles in angiogenesis and lymphangiogenesis, these proteins have been implicated in tumor growth, inflammation, and metastasis in other studies. Aside from the link between integrins and oncogenes [40], one study on high-grade serous ovarian cancer (HGSOC) found the integrins αVβ3 heterodimer to be atypically localized in cell nuclei [41]. Chronic filarial infection has been associated with cancer in several case studies [42,43]. In our study of lymphatic cells, we see decreased expression of the fibronectin ligand and a similar atypical, nuclear localization of the integrin α5 subunit in cells treated with parasite miRNA, suggesting that it may be worth further exploring whether these miRNAs could have effects related to tumorigenic processes.

Secreted molecules from other helminths such as *S. mansoni* have also been found to affect the expression of other cell-to-cell connection proteins in the vascular endothelium and contribute to disease pathology. Intercellular adhesion molecule-1 (ICAM-1) is an essential cellular adhesion protein also important for transendothelial migration of leukocytes. In schistosomiasis, expression of ICAM-1 and its cognate integrin are strongly upregulated and are used by schistosome eggs to adhere to the endothelium of the liver where they eventually form granulomas in the affected patient [44].

Inflammation is another important mediator of disease pathology with filarial infections. Leukocyte circulation throughout blood, lymphatics, tissue, and lymphoid organs is possible with the help of several integrins and, for dendritic cells in particular, PECAM-1 [45]. Our transcriptomic analysis revealed PECAM-1 to be downregulated by parasite miRNAs, and we confirmed reduced protein expression in our *in vitro* studies. However, it is also known that changes to fluid flow and inflammation can disrupt the presentation of a typical monolayer [45]. Therefore, combined with inappropriate lymph flow and inflammation due to the infection, the presence of parasite miRNAs may worsen lymphatic dysfunction. Since microfilariae also produce these miRNAs, the pathology associated with adult worms could be further amplified.

When lymphatic pathology is established, it becomes irreversible even after treatment or death of the filarial parasites [46]. Therefore, the detection and monitoring of filarial infections are important, however, it remains challenging. Traditional diagnostic methods may have limitations in terms of sensitivity and specificity. However, the presence of secreted parasite miRNAs in the host serum or blood plasma offers a promising alternative for detecting and monitoring infections. These circulating miRNAs can serve as potential biomarkers, providing an additional method for infection detection in mammalian or human hosts. Previous studies have demonstrated the presence of specific signatures of *O. ochengi* miRNAs in nodular fluids and in plasma of infected bovines [28,30]; *O. volvulus*-derived miRNAs were also detected in the serum of infected humans [28]. Therefore, secreted parasite miRNAs circulating in the host serum or blood plasma have the potential to be utilized as specific and sensitive biomarkers [47], providing an additional detection method for infection in mammalian or human hosts.

Unraveling the role of parasite miRNAs in parasite-host interactions, pathology, and monitoring of infection and treatment holds great promise for advancing our understanding of the intricate molecular mechanisms involved in filarial infection. Future studies exploring parasite miRNAs in the modulation of specific immune cells may further elucidate mechanisms associated with pathology in filariasis. The transcriptomic analysis and *in vitro* validation presented in this study have revealed the significant impact of two parasite miRNAs on the integrity of the endothelial cellular monolayer, in particular the disruption of cellular adhesion and cell-to-cell connection pathways, which ultimately leads to increased lymphatic vessel permeability and the clinical manifestations observed in LF patients. This work can lead to a better understanding of the role LECs play in inflammation caused by parasites. Indeed, LECs can secrete cytokines and active molecules (such as prostaglandins) that can modulate immune cells, activate lymphocytes, and recruit immune cells to the damaged tissue. Further investigation of other parasite miRNAs and the lymphatic endothelium can shed light on the potential implications of the interconnectedness between parasitic infections and lymphatic dysfunction.

## Materials and methods

### Mammalian cell lines and miRNA transfection

HMVEC-dAd (hLEC) cells (Lonza) were cultured at 37°C and 5% CO_2_ in 75-cm^2^ flasks containing complete tissue culture medium (Microvascular Endothelial Cell Growth Medium-2, EGM-2, Lonza). Cell culture media was changed routinely every 2–3 days and cells transferred to a new flask every 5–7 days.

To determine the effect of parasite miRNAs on human LEC *in vitro*, cells were transfected with miRNA mimics (ThermoFisher), which are chemically modified double-stranded RNA molecules that mimic endogenous miRNAs. First, cells were seeded on 12-well plates and allowed to reach full confluency before beginning each experiment. Then, cells were soaked with a solution containing the *B. malayi* miR-5864 or miR-86 mimics, or a scrambled miRNA sequence to serve as Negative Control 5 (NC, ThermoFisher). Each transfection consisted of 25 nM miRNA in conjunction with 1X Lipofectamine RNAiMax (ThermoFisher) in plain media (Endothelial Cell Base Medium-2, EBM-2, Lonza). Two to four biological replicates per transfection group were used in each experiment. During the transfection period of 4–6 hours, cells were incubated at 37°C and 5% CO_2_. Then, complete media (EGM-2) was added, and cells were returned to the incubator for an additional 1 to 3 days. Cell viability was determined by using live cell staining with POPO-3 iodide (2 µM final concentration, ThermoFisher) for dead cells and Hoechst 33342 (10 µM final concentration, ThermoFisher) for staining all nuclei. The number of POPO-3 iodide-positive (dead) cells was less than 1% (Magnification 20x, 5 fields) 24- and 48-hours post-transfection with miRNA mimics.

### Sequencing and transcriptomic analysis

hLECs were cultured in 12-well plates and allowed to reach full confluency before beginning transfection with 1 µM of either Negative Control 5 or bma-miR-5864 or bma-miR-86 miRNA mimics. Three biological replicates per transfection group were used in each experiment. As above, the transfection period was 4–6 hours in 37°C and 5% CO_2_ conditions. Complete EGM-2 media was added to the cells after the transfection period, and they were returned to the incubator for an additional 24 hours. Total RNA was extracted from the cells using the RNeasy Mini Spin Kit (Qiagen) following manufacturer instructions and used in conjunction with the Illumina Stranded Total RNA Prep with Ribo-Zero Plus kit (Illumina) for library preparation. Libraries were pooled, diluted, and quality-checked according to manufacturer specifications before loading onto a P3 type flow cell (Illumina) to obtain 151nt paired-end reads. Sequencing was done on the Illumina NextSeq2000.

Adaptor sequences and PCR duplicates were removed from paired-end reads using fastp v0.23.2 [48]. Transcripts for *B. malayi* were quantified using Salmon v1.5.0 [49] with the option allow Dovetail to the human reference GrCh38 mRNA and ncRNA reference. Differential expression analyses were done for human genes using DESeq2 v1.44.0 [50] comparing the negative control against bma-miR-5864 and bma-miR-86 mimics independently. Additionally, clusterProfiler v4.12.0 [51] was used to run a KEGG enrichment analysis with the differentially expressed genes using FDR < 0.05.

We used miRanda v3.3a [18] with standard parameters (score > 150 and energy <−20 kjoules, typically used to remove predictions with low probability) to predict which 3’UTRs of human transcripts were potential targets for the bma-miRs selected. Human UTRs were obtained by BioMart [52].

### Extracellular vesicle collection, miRNA extraction, and TaqMan miRNA assays

EVs were collected from cultured adult *B. malayi* worms that were obtained from the Filariasis Research Reagent Resource (FR3) Center for distribution through BEI Resources, NIAID, NIH. At the FR3 facility, the worms were recovered from the peritoneal cavity of infected Mongolian gerbils (*Meriones unguiculatus*) interperitoneally infected with *Brugia malayi*, NR-49238. This study protocol was approved by the Institutional Animal Care and Use Committee at the University of Georgia (Protocol A2022 04-006). Worms were washed in RPMI-1640 media before being shipped. In our laboratory, adult female and male *B. malayi* worms were separated (10 worms/well) into a 6-well plate containing 5 mL/well complete culture media (RPMI-1640, 10% FBS, 1x penicillium/streptomycin mix) and incubated at 37°C and 5% CO_2_. Three wells each were used for males and females. Supernatant from adult worms was collected on Day 2. EVs were isolated from the supernatants following manufacturer instructions for the ExoQuick-TC Ultra EV Isolation Kit for Tissue Culture Media (System Biosciences).

Collected and purified EVs were used to extract miRNA (miRNeasy Mini Spin Kit, Qiagen). Detection of parasite miRNA of interest (bma-miR-5864, bma-miR-86) was done using RT-qPCR with specific TaqMan miRNA Assay (ThermoFisher) probes for bma-miR-5864 and bma-miR-86, or no probes as negative control. Cycle threshold (Ct) values were determined using QuantStudio 6 Pro thermocycler.

### Cell monolayer permeability assays

hLECs were seeded in an insert basket on a 24-well transwell plate following manufacturer instructions for the *In Vitro* Vascular Permeability Assay (Millipore Sigma). Cells were then transfected with bma-miR-5864, bma-miR-86, or NC miRNA mimics, with 8 biological replicates for each transfection group. FITC-Dextran was also added into each insert at the start of the transfection. The fluorescence of FITC was measured in the wells after 24- and 48-hours post transfection. Florescent signal was measured by a microplate reader (Synergy H1, BioTek).

### Protein extraction and western blotting

hLEC proteins were collected following miRNA-mimic transfection using RIPA buffer with 0.1% SDS. Proteins were separated by SDS-PAGE gel electrophoresis and transferred onto a PVDF membrane. Membranes were incubated overnight at 4°C with primary antibodies against the proteins of interest (anti-FN1, anti-ITGA5, anti-PECAM-1, Invitrogen; anti-ITGB1, Sigma) and alpha tubulin (Invitrogen) at a concentration of 1:1000 in TBS-0.1% Tween-20 (Sigma). Secondary antibodies (goat anti-mouse 680RD and goat anti-rabbit 800CW, LiCor) were incubated at room temperature at a concentration of 1:20000 for one hour. Fluorescent signal was detected and measured by a LiCor instrument regardless of brightness or contrast levels, that results in user-independent calculation of signal. Alpha tubulin served as a reference protein to normalize fluorescence signals of the proteins of interest per each sample. Two to three biological replicates were included in each Western blot experiment.

### Immunofluorescence microscopy

hLECs were seeded on 8-well chamber slides 24 hours prior to transfection. To investigate the effects of parasite miRNAs on protein localization, cells were transfected with bma-miR-5864 mimic, bma-miR-86 mimic, or NC mimic as described above. After transfection, complete media was added, and cells were returned to the incubator for 24 hours.

Following transfection, cells were washed with PBS and fixed using 4% formaldehyde (Sigma) in PBS and 0.15% Triton-X (Sigma) in PBS. Primary antibodies were incubated at a concentration of 1:200 in PBS overnight at 4°C. Secondary antibodies (Alexa Fluor 594 (red) or 488 (green), ThermoFisher) were incubated at a concentration of 1:250 in PBS for one hour at room temperature. Approximately 200 µL of mounting medium (VECTASHIELD Vibrance Antifade Mounting Medium with DAPI, Vector Labs) was added to each chamber and allowed to intercalate for one hour at room temperature prior to microscopy. Samples were analyzed using a Leica DMi8 Thunder microscope (Leica) at 40x magnification.

### Statistical analyses

Statistical analyses were performed with PRISM 9.3.1 software (Graph Pad Inc). The graphs present average (mean) values with error bars indicating Standard Deviation. The comparisons between groups were made using non-parametric *t*-tests. A *p*-value < 0.05 was considered to be statistically significant.

## Supporting information

S1 Fig Gaps between cells in bma-miRNA transfected and NC hLEC samples. **Column 1.** Three bright fields of NC hLECs. **Column 2.** Three bright fields of bma-miR-5864 mimic treated hLECs. Stars indicate gaps between cells (examples). **Column 3.** Three bright fields of bma-miR-86 mimic treated hLECs. Stars indicate gaps between cells (examples). Nuclei are shown in blue (DAPI). Bar is 20 µm.
(TIF)

S1 Table MiRanda predicted seed sites of bma-miRs on human 3UTRs. **A.** MiRanda predicted seed sites of bma-miR-5864 on human 3UTRs. Targets selected by energy level below −20 kjoules and score higher than 150. **B.** MiRanda predicted seed sites of bma-miR-86 on human 3UTRs. Targets selected by energy level below −20 kjoules and score higher than 150. Total score, total energy were calculated as summary of scores or energy of all seed sites found on the UTR; max score, min energy indicate maximum and minimum parameters of seed sites found on the UTR.
(XLSX)

S2 Table DEGs in bma-miRs transfected LECs as compared to control cells. **A.** DEGs in bma-miR-5864 transfected LECs as compared to control cells. **B.** DEGs in bma-miR-86 transfected LECs as compared to control cells.
(XLSX)

S3 Table Downregulated genes (adjusted *p*-value < 0.05) in bma-miRs transfected LECs as compared to control. **A.** Downregulated genes (adjusted *p*-value < 0.05) in bma-miR-5864 transfected LECs as compared to control. Selected genes have predicted seed site(s) for bma-miR-5864. **B.** Downregulated genes (adjusted *p*-value < 0.05) in bma-miR-86 transfected LECs as compared to control. Selected genes have predicted seed site(s) for bma-miR-86.
(XLSX)

S4 Table Enrichment analysis of downregulated genes in LECs transfected with bma-miRs as compared to control. **A.** Enrichment analysis of downregulated genes in LECs transfected with bma-miR-5864 as compared to control. In the analysis, the genes that have predicted seed site(s) for the miR were used. **B.** Enrichment analysis of downregulated genes in LECs transfected with bma-miR-86 as compared to control. In the analysis, the genes that have predicted seed site(s) for the miR were used.
(XLSX)
